# Transcatheter versus surgical aortic valve replacement in patients with aortic regurgitation: A propensity-matched analysis

**DOI:** 10.1016/j.heliyon.2023.e16734

**Published:** 2023-05-26

**Authors:** Chi Zhou, Zongyi Xia, Yanxu Song, Zhexun Lian

**Affiliations:** Department of Cardiology, The Affiliated Hospital of Qingdao University, Qingdao, Shandong, 266003, China

**Keywords:** Aortic regurgitation, Transcatheter aortic valve replacement, Surgical aortic valve replacement

## Abstract

This study aimed to analyze in-hospital and early-to-interim outcomes of pure aortic regurgitation (AR) using transcatheter aortic valve replacement (TAVR) vs. surgical aortic valve replacement (SAVR).

**Background:**

Few studies have discussed and compared the safety and short-term prognosis of TAVR and SAVR in pure AR patients. As such, we looked to the National Readmissions Database (NRD) for records between 2016 and 2019 in order to identify patients diagnosed with pure AR who underwent SAVR or TAVR. We used the propensity score matching to minimize disparities between two groups. We included 23,276 pure AR patients: 1983 (8.5%) who underwent TAVR and 21,293 (91.5%) who underwent SAVR. We found 1820 matched pairs using propensity score matching. In the matching cohort, TAVR was associated with a low risk of in-hospital mortality. Although TAVR had lower incidences of 30-day all-cause readmission (hazard ratio (HR):0.73, 95% confidence interval (CI): 0.61–0.87; *P* < 0.01) and 6-month all-cause readmission (HR: 0.81, 95% CI: 0.67–0.97; *P* = 0.03), while TAVR had high incidences of 30-day permanent pacemaker implantation incidence (HR: 3.54, 95% CI: 1.62–7.74; *P* < 0.01) and 6-month permanent pacemaker implantation incidence (HR: 4.12, 95% CI: 1.17–14.4; *P* = 0.03).

In conclusion, TAVR and SAVR had similar risks of hospital death and lower rates of 30-day and 6-month all-cause and cardiovascular readmission. But TAVR had a higher risk of permanent pacemaker implantation than SAVR in AR patients, suggesting that TAVR can be performed safely in pure AR patients.

## 1 Introduction

The occurrence of aortic regurgitation (AR) in the population is approximately 10% [1], and tends to increase gradually with age [2]. The prognosis for patients with severe AR is extremely poor. Dujardin et al. reported 1-year mortality rate in severe AR patients was about 40% who received the conservative treatment [3]. As of now, surgical aortic valve replacement (SAVR) is considered the primary treatment and serves as the preferred course of action for individuals in need of treatment for aortic regurgitation (AR).

In 2002, Cribier et al. successfully performed the first transcatheter aortic valve replacement (TAVR) [4]. TAVR has now been extended to people at high, medium-, and low-risk of aortic stenosis (AS) [[5], [6], [7]]. As the indications for TAVR in patients with severe AS have expanded, some inoperable patients with pure AR, namely patients with AR due to aortic valve insufficiency and without AS, have also undergone TAVR. In the latest guidelines, TAVR has been recommended for patients who are unable to tolerate surgery, given the poor prognosis of severe AR [8].

Most randomized controlled trials focused on TAVR in AS patients, regardless of the existence of AR. Mostly small retrospective clinical studies that have evaluated AR patients undergoing TAVR. Early retrospective results showed safe and effective results of TAVR in AR patient [9]. Studies comparing SAVR and TAVR in AR patients are lacking. Our study used the National Readmission Database (NRD) to analyze the in-hospital and early-to-interim outcomes of SAVR and TAVR in AR patient.

## 2 Methods

### 2.1 Data source

We selected research patients from the NRD between 2016 and 2019. The NRD was created by the Agency for Healthcare Research and Quality, and comprises all-payer datasets for the Healthcare Cost and Utilization Project. Relevant clinical and resource information, including patient basic patient information, hospital information, hospitalization cost, in-hospital mortality, readmission rate. We used the International Classification of Diseases, Tenth Revision (ICD-10) to identify the diagnosis and procedure. This database consists of discharge records for twenty-eight states and represents approximately 35 million weighted annual discharges excluding records from rehabilitation centers and long-term acute care facilities. As this observational study used publicly available data of unidentifiable patients, it was not subject to an ethics committee review. Due to an irregularity in the NRD contact numbers across the years, we elected to exclude patients discharged during December in order to complete the 30-day follow-up. Additionally, the second cohort was selected patients discharged between July and December to complete the 6-month follow-up.

### 2.2 Study population

We searched the NRD from 2016 to 2019 for patients with AR who underwent SAVR or TAVR using ICD-10 codes (AR: I35.1; SAVR: 02RF3; TAVR: 02RF0). We excluded patients aged <18 years, those patients with a history of other cardiac surgeries, and those with missing data. Patients who had previously undergone prosthetic valve implantation or had infective endocarditis, which reduced their likelihood of being suitable candidates for SAVR were excluded. Patients who diagnosed with AS were excluded encoded by ICD-10 (AR: I350, I352).

### 2.3 Outcomes

The study aimed to investigate the in-hospital outcomes, namely in-hospital mortality, mechanical ventilation, transfusion, sepsis, bacterial pneumonia, acute kidney injury (AKI), cardiac arrest, and intracranial hemorrhage, as the primary endpoints. The secondary endpoints comprised 30-day and 6-month follow-up outcomes, including cardiovascular (CV) readmission, heart failure (HF) readmission, all-cause readmission, and pacemaker implantation. CV readmission was operationally defined as hospitalization related to myocardial infarction, arrhythmia, or HF. Supplementary Table 1 listed the ICD-10 codes utilized in the present study. The primary diagnosis leading to 30-day and 6-month readmissions was identified as the reason for readmission. Patients with AR who underwent TAVR or SAVR were matched using propensity scores in a 1:1 ratio.

### 2.4 Statistical analyses

The means and standard deviations of continuously distributed variables following a normal distribution are reported as mean ± standard deviation and were analyzed using Student's t-test. In contrast, non-normally distributed continuous variables are reported as the median (interquartile range [IQR]) and analyzed using a Wilcoxon rank-sum test. Categorical variables were expressed in percentages, and comparisons between groups were achieved utilizing either X^2^ test or Fisher's exact test. Univariate logistic regression analysis was performed to calculate the odds ratio (OR) accompanied by the 95% confidence interval (CI) for in-hospital outcomes.

**Fig. 1:**
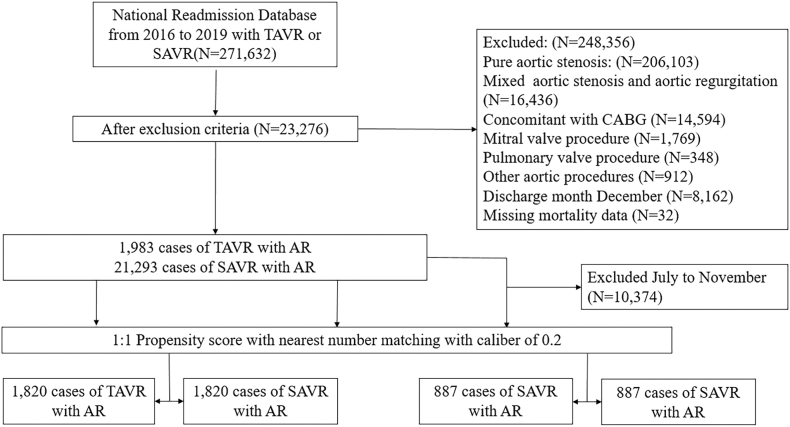
Diagram of patient selection process. Abbreviation: SAVR: surgical aortic valve replacement; AR: aortic regurgitation; TAVR: transcatheter aortic valve replacement.

We chose 1:1 nearest-neighbor matching to find the closest propensity score matching (PSM) between TAVR and SAVR. The maximum allowable difference in the propensity score (caliper width) between paired observations was 0.2 in various models to maintain adequate sample size and balance between the groups. The matching R package was used for analysis. The variables used in the PSM included age, female sex,

The variables used in PSM include the following: (1) general condition, including age, sex, smoking, obesity, income, pay, elective, alcohol use; (2) history of cardiac treatment: percutaneous coronary intervention (PCI), permanent pacemaker implantation (PPM), coronary artery bypass graft (CABG). (3) comorbidity: congestive HF, chronic pulmonary disease, peripheral vascular disease, myocardial infarction, peptic ulcer disease, anemia, dyslipidemia, diabetes, renal disease, liver disease, prior stroke, rheumatic disease, paraplegia, dementia, atrial fibrillation, cerebrovascular disease, Charlson Comorbidity Index and metastatic solid tumor. Supplementary Fig. 1 shows the data distribution before and after propensity matching. Fig. 1 illustrates the selection process.

The univariate Cox proportional hazards regression was used to calculate the hazard ratios (HRs) and 95% CI for the 30-day and 6-month outcomes. Additionally, Kaplan-Meier (K-M) curves were plotted for 30-day and 6-month all-cause readmission. *P* < 0.05 as statistical significance, we used R 4.0.2 as statistical analyses software.

## 3 Results

### 3.1 Characteristics of the primary cohort

Between 2016 and 2019, 23,276 patients underwent SAVR or TAVR for AR including 21,293 (91.5%) and 1983 (8.5%) patients who underwent SAVR and TAVR, respectively. After PSM, we included 1820 matched pairs with 30-day follow-up. In the Supplementary Fig. 2 showed the trends of AR patients receiving TAVR or SAVR between 2016 and 2019.

Before matching, TAVR group average age was 70 years, which was higher than the average age in the SAVR group (58 years). Female patients accounted for 35% of all TAVR patients and 27% of SAVR patients. Patients who underwent TAVR had more comorbidities, such as congestive HF, chronic myocardial infarction, dementia, anemia, history of cancer, liver disease and renal disease. The TAVR group exhibited a higher probability of prior cardiac surgery including PCI, CABG and PPM. The SAVR group had higher proportions of patients with peripheral vascular disease and obesity. No differences were observed in rheumatic disease, paraplegia, or alcohol use between the two groups. The TAVR group had a higher Charlson Comorbidity Index, indicating a heavier comorbidity burden.

### 3.2 Characteristics of the matching cohort

After PSM, we included 1820 matched pairs in the matched cohort and 887 matched pairs in the second cohort. Table 1 showed the detailed baseline distinctions between the two groups in the primary and matching cohorts. Moreover, Supplementary Table 2 summarizes in detail the baseline distinctions between the two groups in the second and matching cohorts. The data distribution before and after PSM matching in the second cohort was showed in the Supplementary Fig. 3.

**Table 1:** Baseline characteristics of the study population who underwent TAVR and SAVR before and after matching.

Variable	Primary cohort			Matching cohort		
	SAVR	TAVR	*P-*valve	SAVR	TAVR	*P-*valve
Number	21,293	1983		1820	1820	
Age	58.88 ± 13.69	73.47 ± 11.81	<0.001	69.98 ±10.41	70.49 ± 12.49	0.179
Female	5919 (27.8%)	756 (38.1%)	<0.001	650 (35.7%)	655 (36.0%)	0.890
Elective	5749 (27.0%)	535 (27.0%)	0.148	499 (27.4%)	512 (28.1%)	0.657
Median income range^a^			0.456			0.062
0-25th percentile	4216 (19.8%)	389 (19.6%)		352 (19.3%)	366 (20.0%)	
25-50th percentile	5259 (24.7%)	494 (24.9%)		483 (26.5%)	416 (23.3%)	
50-75th percentile	5898 (27.7%)	531 (26.8%)		510 (28.0%)	500 (27.5%)	
75-100th percentile	5898 (27.7%)	569 (28.7%)		475 (26.1%)	538 (29.2%)	
Bed size^b^			<0.001			0.688
Small	1213 (5.7%)	61 (3.1%)		61 (3.4%)	53 (2.9%)	
Medium	4281 (20.1%)	359 (18.1%)		326 (17.9%)	337 (18.5%)	
Large	15,799 (74.2%)	1563 (78.8%)		1433 (78.7%)	1430 (78.6%)	
Pay			<0.001			0.491
Medicare	8049 (37.8%)	1559 (78.6%)		1322 (72.6%)	1334 (73.3%)	
Medicaid	2193 (10.3%)	76 (3.8%)		103 (5.7%)	87 (4.8%)	
Others	11,051 (51.9%)	348 (17.6%)		395 (21.7%)	399 (21.9%)	
Prior PCI	767 (3.6%)	313 (15.8%)	<0.001	212 (11.6%)	206 (11.3%)	0.795
Prior CABG	383 (1.8%)	299 (15.1%)	<0.001	213 (11.7%)	202 (11.1%)	0.602
Prior PPM	617 (2.9%)	216 (10.9%)	<0.001	186 (10.2%)	177 (9.7%)	0.658
Smoke	6324 (29.7%)	724 (36.5%)	<0.001	616 (33.8%)	611 (33.6%)	0.888
Dyslipidemia	10,156 (47.7%)	1307 (65.9%)	<0.001	1102 (60.5%)	1110 (61.0%)	0.812
Anemia	788 (3.7%)	115 (5.8%)	0.001	84 (4.6%)	85 (4.7%)	1.000
Obesity	4535 (21.3%)	359 (18.1%)	0.001	324 (17.8)	327 (18.0)	0.931
Alcohol use	64 (0.3%)	NA	0.521	NA	NA	1.000
Chronic pulmonary disease	4936 (17.0%)	535 (27.0%)	<0.001	489 (26.9%)	486 (26.7%)	0.940
Prior stroke	1837 (6.3%)	238 (12.0%)	<0.001	211 (11.6%)	213 (11.7%)	0.959
Congestive heart failure	8599 (40.2%)	1501 (75.7%)	<0.001	1326 (72.9%)	1334 (73.3%)	0.794
Dementia	85 (0.4%)	58 (2.9%)	<0.001	32 (1.8%)	39 (2.1%)	0.472
Peptic ulcer disease	362 (2.4%)	87 (4.4%)	<0.001	90 (4.9%)	86 (4.7%)	0.817
Myocardial infarction	1341 (6.5%)	280 (14.1%)	<0.001	237 (13.0%)	232 (12.7%)	0.843
Diabetes	2981 (14.0%)	367 (18.5%)	0.002	282 (15.5%)	287 (15.8%)	0.855
PVD	8960 (41.9%)	506 (25.5%)	<0.001	439 (24.1%)	465 (25.5%)	0.338
Cerebrovascular disease	1405 (6.6%)	188 (9.5%)	0.001	159 (8.7%)	153 (8.4%)	0.767
Renal disease	2662 (12.5%)	348 (30.6%)	<0.001	505 (27.7%)	509 (28.0%)	0.912
Rheumatic disease	100 (0.3%)	16 (0.8%)	0.335	11 (0.6%)	NA	0.823
Metastatic solid tumor	43 (0.2%)	18 (0.9%)	<0.001	23 (1.3%)	16 (0.9%)	0.334
Paraplegia	277 (1.3%)	16 (0.8%)	0.006	13 (0.7%)	13 (0.7%)	1.000
Liver disease	681 (3.2%)	87 (4.9%)	<0.001	96 (5.3%)	95 (5.2)	1.000
Charlson comorbidity index	4.10 ± 2.09	6.33 ± 2.15	<0.001	5.95± 2.16	5.97 ± 2.22	0.705

To express normally distributed continuous variables, we utilized the mean value ± standard deviation and conducted analysis via Student's t-test. Meanwhile, categorical variables were expressed numerically with percentages using either the X^2^ test or Fisher's exact test.

NA: mean < 11 numbers are not reported according to the database suggestion.

Abbreviation: TAVR: transcatheter aortic valve replacement; SAVR: surgical aortic valve replacement; PCI: percutaneous coronary intervention; CABG: coronary artery bypass graft; PPM: permanent pacemaker implant; PVD: peripheral vascular disease.

a The median income in the ZIP code of each patient was stratified into quartile divisions annually, and thus categorized into groups representing low income (bottom 25%), medium income, high income, and the highest income (top 25%).

b The categorization of hospital sizes was predicated upon the total number of hospital beds, with designated cut-points being established for every combination of region and location. This was done in such a manner that roughly 1/3 of hospitals were classified within each size category.

### 3.3 In-hospital outcomes

In the matched group, there were no differences in the in-hospital mortality (3.2% vs. 2.1%; *P* = 0.064, OR: 0.66, 95% CI: 0.44–1.00). TAVR group exhibited a reduced risk of acute kidney injury, mechanical ventilation, transfusion, and bacterial pneumonia than SAVR group. Furthermore, the SAVR group had a longer hospital stay (8 vs. 3 days, *P* < 0.001) and greater cost (50,456 vs. 45,310$, *P* < 0.001) than the TAVR group. The risk of stroke, sepsis, cardiac arrest, intracranial hemorrhage, and pacemaker implantation did not show difference between the two groups. Table 2 illustrated the detailed hospital outcome differences between the two groups in the primary and matched cohorts. Table 3 illustrated the odds ratio between the two groups in the matched cohort.

**Table 2:** In-hospital outcomes of the study population who underwent TAVR and SAVR before and after matching.

Variable	Primary cohort			Matching cohort		
	SAVR	TAVR	*P*-value	SAVR	TAVR	*P*-value
Number	21,293	1983		1820	1820	
In hospital death	443 (2.1%)	40 (2.0%)	0.914	58 (3.2%)	39 (2.1%)	0.064
PPM	1064 (5.0%)	202 (10.2%)	<0.001	123 (6.8%)	195 (10.7%)	<0.001
Transfusion	3622 (17.0%)	119 (6.0%)	<0.001	391 (21.5%)	115 (6.3%)	<0.001
Pneumonia bacterial	958 (4.5%)	54 (2.7%)	<0.001	96 (5.3%)	54 (3.0%)	0.001
Mechanical ventilation	617 (2.9%)	40 (2.0%)	0.028	57 (3.1%)	40 (2.2%)	0.099
Intracranial hemorrhage	180 (0.8)	NA	0.027	14 (0.8%)	NA	0.189
Sepsis	1290 (6.1%)	43 (2.2%)	<0.001	102 (5.6)	42 (2.3%)	<0.001
Acute kidney injury	3938 (18.5%)	342 (17.2%)	0.179	502 (27.6%)	312 (17.1%)	<0.001
Cardiac arrest	199 (0.9%)	17 (0.9%)	0.825	21 (1.2%)	17 (0.9%)	0.623
Stroke	804 (3.8%)	37 (1.9%)	<0.001	69 (3.8%)	37 (2.0%)	0.002
Length of stay, median (IQR), day	7.5 [5,13]	3 [2,9]	<0.001	8 [5,13]	3 [1,7]	<0.001
Cost, median (IQR), USD^a^	44,790 [29,960, 69,300]	44,320 [30,227, 67,863]	0.96	50,456 [33,741, 79,102]	45,310 [30,430, 69,672]	<0.001

Non-normally distributed continuous variables were expressed by medians (interquartile range [IQR]) whilst being analyzed via the Wilcoxon rank-sum test. Conversely, categorical variables were expressed numerically with percentages and analyzed with either the Fisher's exact test or X^2^ test.

NA: mean < 11 numbers are not reported according to the database suggestion.

Abbreviation: TAVR: transcatheter aortic valve replacement; PPM: permanent pacemaker implant; IQR: interquartile range; USD: United States dollar; SAVR: surgical aortic valve replacement; PCI: percutaneous coronary intervention; CABG: coronary artery bypass graft.

a Adjusted for inflation to 2019.

**Table 3:** The in-hospital odds ratio outcomes for TAVR versus SAVR in the matching cohort.

Variable	OR(95%CI)	*P*-value
In-hospital mortality	0.66(0.44–1.00)	0.52
Transfusion	0.25(0.20–0.31)	<0.01
Sepsis	0.40(0.27–0.57)	<0.01
Stroke	0.53(0.35–0.78)	<0.01
Acute kidney injury	0.54(0.46–0.64)	<0.01
Mechanical ventilation	0.69(0.46–1.04)	0.08
Pneumonia bacterial	0.55(0.39–0.77)	<0.01
Permanent pacemaker implantation	1.65(1.31–2.10)	<0.02

Univariate logistic regression was used to calculate the OR with a 95% confidence interval for in-hospital outcomes.

Abbreviation: SAVR: surgical aortic valve replacement; OR: Odds ratio; TAVR: transcatheter aortic valve replacement; CI: confidence interval.

### 3.4 30-Day readmissions etiologies

**Fig. 2:**
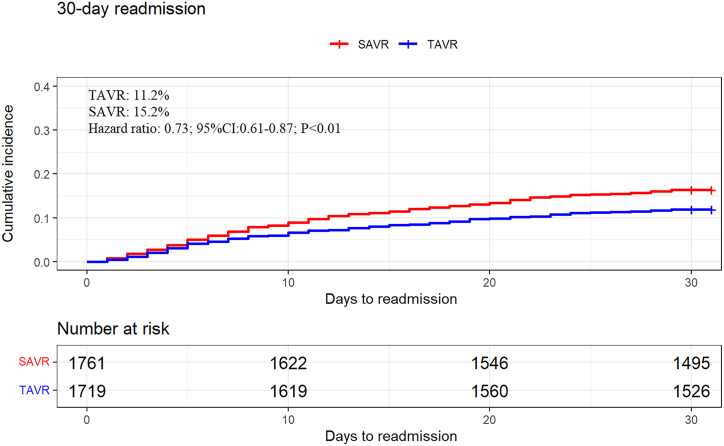
K-M curves show the 30-day all-cause readmission rates comparing SAVR with TAVR in pure AR patients after matching. Univariate Cox proportional hazards regression to calculate hazard ratio and 95% CI for the 30-day all-cause readmission. Abbreviation: K–M: Kaplan-Meier; TAVR: transcatheter aortic valve replacement; SAVR: surgical aortic valve replacement; AR: aortic regurgitation; CI: confidence interval.

**Fig. 3:**
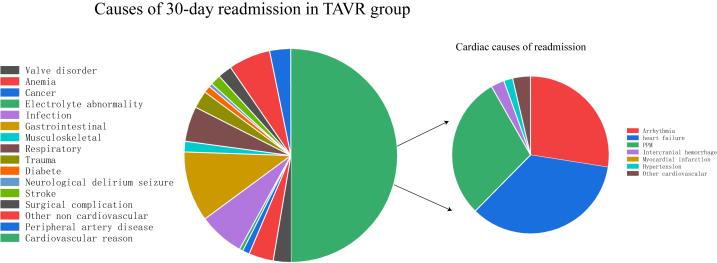
The etiologies of 30-day readmission after TAVR. Abbreviation: TAVR: AR: aortic regurgitation; transcatheter aortic valve replacement; PPM: permanent pacemaker implant.

**Fig. 4:**
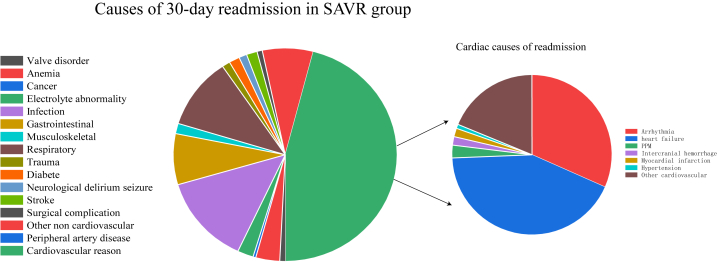
The etiologies of 30-day readmission after SAVR. Abbreviation: SAVR: surgical aortic valve replacement; AR: aortic regurgitation; PPM: permanent pacemaker implant.

In the matched cohort, cardiac cause is the most common reason for readmission for TAVR and SAVR. HF (18.7%), PPM (14.8%), and arrhythmias (15.7%) were the most common cardiac causes in the TAVR group. Heart failure (19.5%), arrhythmias (14.4%), and other CV (15.7%) were the most common cardiac causes in the SAVR group. Compared with TAVR patients, SAVR patients had a higher rate of readmission for respiratory and infectious reasons. The K-M curves of 30-day all-cause readmission are shown in Fig. 2. Fig. 3, Fig. 4 show the etiologies of 30-day readmission after TAVR and SAVR, respectively. The 30-day HR outcomes for TAVR versus SAVR in the matching cohort are shown in Table 4. The detailed 30-day readmission etiologies is shown in Supplementary Table 3.

**Table 4:** The 30-day hazard ratios outcomes for TAVR versus SAVR in the matching cohort.

Variable	HR(95% CI)	*P*-value
30-day outcome		
Cardiovascular readmission	0.58(0.44–0.77)	<0.01
All-cause readmission	0.73(0.61–0.87)	<0.01
Heart failure readmission	0.60(0.33–1.08)	0.09
Stroke readmission	0.97(0.31–3.02)	0.96
Permanent pacemaker implantation readmission	3.54(1.62–7.74)	<0.01

Univariate Cox proportional hazards regression to calculate HR and 95% CI for the 30-day outcomes.

Abbreviation: SAVR: surgical aortic valve replacement; CI: confidence interval; TAVR: transcatheter aortic valve replacement; HR: hazards ratios.

### 3.5 6-Month readmissions etiologies

**Fig. 5:**
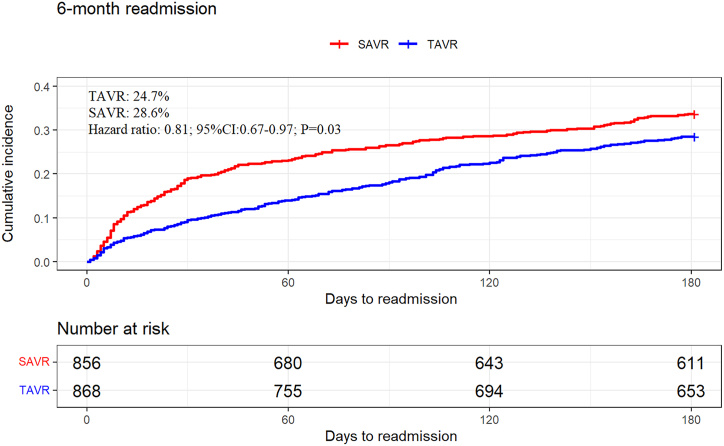
K-M curves show the 6-month all-cause readmission rates comparing SAVR with TAVR in pure AR patients after matching. Univariate Cox proportional hazards regression to calculate hazard ratio and 95% CI for the 6-month all-cause readmission. Abbreviation: K–M: Kaplan-Meier; SAVR: surgical aortic valve replacement; AR: aortic regurgitation; TAVR: transcatheter aortic valve replacement; CI: confidence interval.

**Fig. 6:**
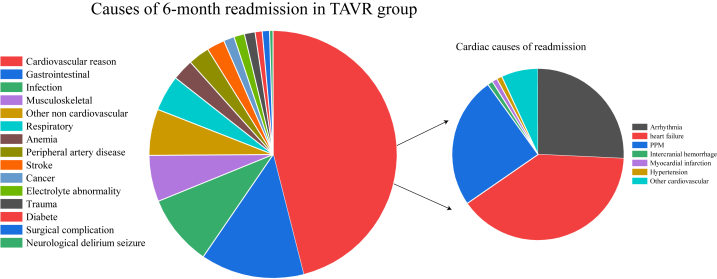
The etiologies of 6-month readmission after TAVR. Abbreviation: AR: aortic regurgitation; TAVR: transcatheter aortic valve replacement; PPM: permanent pacemaker implant.

**Fig. 7:**
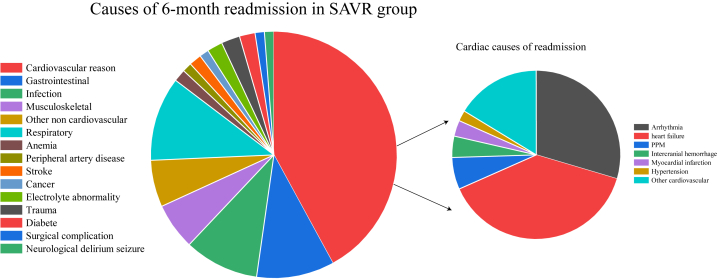
The etiologies of 6-month readmission after SAVR. Abbreviation: AR: aortic regurgitation; SAVR: surgical aortic valve replacement; PPM: permanent pacemaker implant.

In the matched cohort, cardiac cause is the most common reason for readmission for TAVR and SAVR. HF (19.7%), arrhythmias (12.0%), and PPM (11.6%) were the most common cardiac causes in the TAVR group. HF (14.7%), arrhythmias (11.2%), and other CV (6.2%) were the most common cardiac causes in the SAVR group. Compared with SAVR patients, TAVR patients have a lower rate of readmission for respiratory reasons. The K-M curves of 6-month all-cause readmission are shown in Fig. 5. Fig. 6, Fig. 7 show the etiologies of 6-month readmission after TAVR and SAVR. In Table 5 shows the 6-month HR outcomes for TAVR versus SAVR. The detailed 6-month readmission etiologies in the Supplementary Table 4. In the Supplementary Fig. 4, compared TAVR and SAVR in 30-day all-cause readmission incidence, 6-month all-cause readmission incidence, 30-day permanent pacemaker implantation incidence, and 6-month permanent pacemaker implantation incidence.

**Table 5:** The 6-month hazard ratios outcomes for TAVR versus SAVR in the matching cohort.

Variable	HR(95% CI)	*P*-value
6-month outcome^a^		
Cardiovascular readmission	0.58(0.42–0.78)	<0.01
All-cause readmission	0.81(0.67–0.97)	0.03
Heart failure readmission	0.73(0.36–1.46)	0.37
Stroke readmission	0.75(0.20–2.78)	0.66
Permanent pacemaker implantation readmission	4.12(1.17–14.46)	<0.01

Univariate Cox proportional hazards regression to calculate HR and 95% CI for the 6-month outcomes.

Abbreviation: SAVR: surgical aortic valve replacement; CI: confidence interval; HR: hazards ratios; TAVR: transcatheter aortic valve replacement.

a The 6-month outcome based on the second cohort after matching.

## 4 Discussion

We studied the in-hospital and early-to-interim outcomes of TAVR and SAVR in AR patients. The main findings from our research are as follows: (1) Over study period, there was a noticeable increase in the proportion of TAVR patients who underwent aortic valve replacement for pure AR. (2) TAVR had a comparable in-hospital mortality rate to SAVR but had a lower incidence of postoperative complications, including stroke, mechanical ventilation, pleural effusion, bacterial pneumonia and acute kidney injury. (3) Patients with pure AR who underwent SAVR had a longer hospital stay and higher costs than those who underwent TAVR. (4) In terms of 30-day and 6-month outcomes, compared to TAVR, SAVR had a higher risk of all-cause readmission and CV readmission and similar stroke and HF readmission rates but had a lower risk of pacemaker implantation. In well-known randomized controlled studies comparing TAVR and SAVR, such as the PARTNER study, patients with pure AR are often excluded. Few observational studies have shown that TAVR is safe for use in patients with AR. The lack of high-quality clinical evidence has limited utilization of TAVR in AR patients.

In a retrospective study based on the Germany Institute for the Hospital Remuneration System from 2008 to 2015, approximately 6.5% of patients with AR were treated with TAVR [10]. Alharbi*et al.* used national inpatient sample and found that approximately 6.2% of AR patients underwent TAVR [11]. In our study, approximately 8% of AR patients underwent TAVR, which is higher than that reported in previous studies; potentially because our study is more recent than the previous study. In 2019, the United States Food and Drug Administration approved TAVR for low-risk surgical patients. The expansion of TAVR indications has led to a noticeable rise in the number of TAVR.

Before matching, pure AR patients in the TAVR group had more comorbidities and were older. In our study, before and after matching, TAVR and SAVR groups had similar risks of in-hospital mortality. Despite the absence of a statistical disparity seen in the in-hospital mortality rate exhibited between the two groups in the matching cohort, SAVR demonstrated a relatively higher numerical value (3.2% vs. 2.1%, *P* = 0.064). A previous study conducted by Alharbiet et al. found no difference in-hospital mortality rate between two groups in pure AR (3.2% vs. 2.7%; *P* = 0.49). We reported a 2% in-hospital mortality rate, which is lower the 3% in-hospital mortality rate reported in the Aortic Regurgitation (AR-TAVR) study [12]. This may be related to technological advances in TAVR and the proportions of the low-risk population. This suggests the in-hospital mortality rate in AR patient who underwent TAVR is not lower than that in those who underwent SAVR.

TAVR is a less invasive procedure that allows faster recovery in patients with AR. Similar in previous AS studies [13,14], we also found that TAVR had fewer surgical complications, including acute kidney injury, mechanical ventilation, pleural effusion, and bacterial pneumonia. Furthermore, we also reported that approximately 10% of patients received PPM after TAVR, which was higher than that after SAVR. The incidence of postoperative PPM in our retrospective study was consistent with that in a previous meta-analysis by Wernly et al. [15]. Additionally, our study also analyzed the 30-day and 6-month outcomes of PPM after discharge, and the AR patients in the TAVR group had a higher rate of PPM than those in the SAVR group. Liu et al. included 134 high risk patients with severe AR and found that 8.9% of them had a pacemaker implanted after 1 year of follow-up [16].

Regarding the30-day and 6-month outcomes, TAVR group had a lower risk of all-cause readmission and CV readmission rates than SAVR group in matching cohort. Furthermore, TAVR was linked to an increased higher risk of PPM than SAVR. In the primary cohort, there were no statistically significant differences between the two groups in all-cause and CV readmission rates at 30-day and 6-month follow-up. Meanwhile, no significant differences were seen between the groups in hospital costs in the primary cohort. Despite these findings, further investigations are necessary to substantiate these disparities.

## 5 Limitation

Our study had the following limitations. The NRD used for this analysis is an administrative database, and patients were identified through the utilization of ICD-10 codes, who may suffer from coding errors. Although the procedure code was used to identify patients in our cohort, we expected errors in the coding to be negligible. Second, NRD lacks aortic root measurement data (including the aortic annulus length, aortic valve types, aortic annulus area, leaflet calcification, annular calcification and calcification volume) and preoperative ultrasound data (including the effective aortic valve orifice area, ejection fraction, pulmonary artery pressure and left ventricular diameter). We could not obtain the Society of Thoracic Surgeons risk scores or intraoperative situations including sheath size, valve size, valve type and balloon size. These unmeasured confounding factors may have influenced the results of the analysis. The causes of pure AR in our cohort study could not be determined from the NRD. Third, this study was a retrospective analysis, which has inherent disadvantages. For the 6-month outcomes, we excluded outcomes between July and December of each year, which may have caused a bias. However, randomized grouping cannot be performed as in randomized controlled studies. We used PSM to reduce differences between the two groups. Fourth, the NRD database did not include emergency-related or out-of-hospital deaths. The study only included patients admitted to the hospital in the same state. However, readmissions are uncommon in aortic valve replacement patients. We used the ICD-10 code to identify outcomes, as in other studies that used the Medicare database. It is possible that the coding for some patients was incorrect. However, the frequency of a given outcome being miscoded was similar in a particular group. The large sample size and multicenter retrospective design are this study strength.

## 6 Conclusion

Our study showed an increasing percentage of AR patients are undergoing TAVR. Although patients undergoing SAVR and TAVR have similar in-hospital mortality rates and low postoperative complications, TAVR patients have high PPM rates. TAVR had lower all-cause and CV readmission rates than SAVR in the matching cohort. However, TAVR patients have a high rate of PPM. We believe that TAVR in pure AR patients is safe and can reduce patient readmission rates compared to SAVR. In the future, more patients with pure AR may choose to undergo TAVR.

## Author contribution statement

Chi Zhou: Conceived and designed the experiments; Performed the experiments; Contributed reagents, materials, analysis tools or data; Wrote the paper.

Zongyi Xia: Performed the experiments.

Yanxu Song: Analyzed and interpreted the data; Contributed reagents, materials, analysis tools or data.

Zhexun Lian: Conceived and designed the experiments; Wrote the paper.

## Data availability statement

Data will be made available on request.

## Declaration of ethics statement

The experimental protocol was established, according to the ethical guidelines of the Helsinki Declaration and was approved by the Human Ethics Committee of The Affiliated Hospital of Qingdao University (ID: QYFY WZLL 27355).

## Declaration of competing interest

The authors declare that they have no known competing financial interests or personal relationships that could have appeared to influence the work reported in this paper.

## References

[1] Singh J.P. (1999). Prevalence and clinical determinants of mitral, tricuspid, and aortic regurgitation (the Framingham Heart Study). Am. J. Cardiol..

[2] Fox E.R. (2007). Epidemiology of pure valvular regurgitation in the large middle-aged African American cohort of the atherosclerosis risk in communities study. Am. Heart J..

[3] Dujardin K.S. (1999). Mortality and morbidity of aortic regurgitation in clinical practice. A long-term follow-up study. Circulation.

[4] Cribier A. (2002). Percutaneous transcatheter implantation of an aortic valve prosthesis for calcific aortic stenosis: first human case description. Circulation.

[5] Leon M.B. (2010). Transcatheter aortic-valve implantation for aortic stenosis in patients who cannot undergo surgery. N. Engl. J. Med..

[6] Smith C.R. (2011). Transcatheter versus surgical aortic-valve replacement in high-risk patients. N. Engl. J. Med..

[7] Popma J.J. (2019). Transcatheter aortic-valve replacement with a self-expanding valve in low-risk patients. N. Engl. J. Med..

[8] Vahanian A. (2022). 2021 ESC/EACTS Guidelines for the management of valvular heart disease. Eur. Heart J..

[9] Silaschi M. (2018). The JUPITER registry: one-year outcomes of transapical aortic valve implantation using a second generation transcatheter heart valve for aortic regurgitation. Cathet. Cardiovasc. Interv..

[10] Stachon P. (2020). Nationwide outcomes of aortic valve replacement for pure aortic regurgitation in Germany 2008-2015. Cathet. Cardiovasc. Interv..

[11] Alharbi A.A. (2020). Transcatheter aortic valve replacement vs surgical replacement in patients with pure aortic insufficiency. Mayo Clin. Proc..

[12] Hira R.S. (2017). Trends and outcomes of off-label use of transcatheter aortic valve replacement: insights from the NCDR STS/ACC TVT registry. JAMA Cardiol..

[13] Zack C.J. (2017). Comparative outcomes of surgical and transcatheter aortic valve replacement for aortic stenosis in nonagenarians. Am. J. Cardiol..

[14] Vejpongsa P. (2017). Early readmissions after transcatheter and surgical aortic valve replacement. Cathet. Cardiovasc. Interv..

[15] Wernly B. (2019). Transcatheter aortic valve replacement for pure aortic valve regurgitation: “on-label” versus “off-label” use of TAVR devices. Clin. Res. Cardiol..

[16] Liu L. (2022). One-year outcome after transcatheter aortic valve replacement for aortic regurgitation: a single-center study. J. Card. Surg..

