# Supplementary material for: Transcatheter versus surgical aortic valve replacement in patients with aortic regurgitation: A propensity-matched analysis
Source: Heliyon. 2023 May 26;9(6):e16734. doi: 10.1016/j.heliyon.2023.e16734 (PMC10248226; doi:10.1016/j.heliyon.2023.e16734)

Supplementary Material

|  | | ICD 10 CODE | |
| --- | --- | --- | --- |
| transcatheter aortic valve replacement | 02RF3 | | |
| surgical aortic valve replacement | 02RF0, 02RF4 | |  |
| Atrial fibrillation | I48 | |  |
| Coronary artery bypass graft | 0210-0213 | |  |
| Infective endocarditis | B376,A3282,A3951,A5203,B3321,I330,I339,I38 ,I39 | |  |
| Prior coronary artery bypass graft | Z951 | |  |
| Prior percutaneous coronary intervention | Z955,Z9861 | |  |
| Prior permanent pacemaker implant | Z950,Z95810 | |  |
| Respiratory complications | J956-J958 | |  |
| Transfusion | 3024, 3023 | | |
| Acute kidney injury | N17, N19, N990, R34, R944 | | |
| Cardiac arrest | I46, I469, I462, I9712, I9771, I97710, I97711, I97121 | | |
| Permanent pacemaker | 02HK3JZ,0JH634Z,[02H63JZ](http://www.icd10data.com/ICD10PCS/Codes/0/2/H/6/02H63JZ),[02H60JZ](http://www.icd10data.com/ICD10PCS/Codes/0/2/H/6/02H60JZ),0JH635Z, 02HN4JZ,[02H63NZ](http://www.icd10data.com/ICD10PCS/Codes/0/2/H/6/02H63NZ),[02HK0JZ](http://www.icd10data.com/ICD10PCS/Codes/0/2/H/K/02HK0JZ),[02H60NZ](http://www.icd10data.com/ICD10PCS/Codes/0/2/H/6/02H60NZ),0JH607Z, [02HK3NZ](http://www.icd10data.com/ICD10PCS/Codes/0/2/H/K/02HK3NZ),02H63JZ,JH634Z,[02H64NZ](http://www.icd10data.com/ICD10PCS/Codes/0/2/H/6/02H64NZ),0JH635Z,[02HK4NZ](http://www.icd10data.com/ICD10PCS/Codes/0/2/H/K/02HK4NZ),[02HK4JZ](http://www.icd10data.com/ICD10PCS/Codes/0/2/H/K/02HK4JZ),0JH637Z,0JH605Z,[02HK0NZ](http://www.icd10data.com/ICD10PCS/Codes/0/2/H/K/02HK0NZ),02HN0JZ,[02HK3JZ](http://www.icd10data.com/ICD10PCS/Codes/0/2/H/K/02HK3JZ),[0JH606](http://www.icd10data.com/ICD10PCS/Codes/0/J/H/6/0JH606Z)Z,[02H64JZ](http://www.icd10data.com/ICD10PCS/Codes/0/2/H/6/02H64JZ),0JH636Z,0JH604Z | | |
| Acquired pneumonia | J13-J18 | | |
| Sepsis | T814, T827,T835, T8357, A41, A40, R6520, T8351XA,T80219A,T80211A, B377 | | |
| Mechanical ventilation | 5A1955Z | | |

Supplementary table 1: ICD-10 codes used in our study

Supplementary table 2: Baseline characteristics of the study population who underwent TAVR and SAVR before and after matching in the second cohort.

| Variable | Primary cohort | | | Matching cohort | | |
| --- | --- | --- | --- | --- | --- | --- |
|  | SAVR | TAVR | *P*-value | SAVR | TAVR | *P*-value |
| Number | 11,918 | 984 |  | 887 | 887 |  |
| Age | 58.34 ±13.89 | 71.50 ±12.71 | <0.001 | 70.10 ±10.39 | 70.86 ±12.60 | 0.166 |
| Female | 3,175 (26.6%) | 344 (35.0%) | <0.001 | 343 (38.7%) | 312 (35.2%) | 0.140 |
| Elective | 3,335 (28.0%) | 295 (30.0%) | 0.193 | 284 (32.0%) | 266 (30.0%) | 0.383 |
| Median income range* |  |  | 0.247 |  |  | 0.527 |
| 0-25th percentile | 2,406 (20.1%) | 184 (18.7%) |  | 170 (19.4%) | 169 (19.4) |  |
| 25-50th percentile | 2,924 (24.5%) | 223 (22.7%) |  | 229 (26.2%) | 202 (23.2) |  |
| 50-75th percentile | 3,322 (27.9%) | 290 (29.5%) |  | 238 (27.2%) | 250 (28.7%) |  |
| 75-100th percentile | 3,266 (27.4%) | 287 (29.2%) |  | 238 (27.2%) | 249 (28.6%) |  |
| Bed size** |  |  | <0.001 |  |  | 0.272 |
| Small | 687 (5.8%) | 21 (2.1%) |  | 30 (3.4%) | 19 (2.1%) |  |
| Medium | 2,411 (20.2%) | 162 (16.5%) |  | 148 (16.7%) | 154 (17.4%) |  |
| Large | 8,820 (74.0%) | 801 (81.4%) |  | 709 (79.9%) | 714 (80.5%) |  |
| Pay |  |  | <0.001 |  |  | 0.69 |
| Medicare | 4,338 (36.4%) | 730 (74.2%) |  | 640 (72.2%) | 642 (72.4%) |  |
| Medicaid | 1,253 (10.5%) | 47 (4.8%) |  | 54 (6.1%) | 46 (5.2%) |  |
| Others | 6,327 (53.1%) | 207 (21.0%) |  | 193 (21.8%) | 199 (22.4%) |  |
| Prior PCI | 399 (3.3%) | 130 (13.2%) | <0.001 | 108 (12.2%) | 94 (10.6%) | 0.331 |
| Prior CABG | 223 (1.9%) | 146 (14.8%) | <0.001 | 109 (12.3%) | 98 (11.0%) | 0.460 |
| Prior PPM | 341 (2.9%) | 109 (11.1%) | <0.001 | 98 (11.0%) | 87 (9.8%) | 0.437 |
| Smoke | 3,517 (29.5%) | 354 (36.0%) | <0.001 | 317 (35.7%) | 309 (34.8%) | 0.728 |
| Dyslipidemia | 5,493 (46.1%) | 611 (62.1%) | <0.001 | 521 (58.7%) | 542 (61.1%) | 0.333 |
| Anemia | 419 (3.5%) | 51 (5.2%) | 0.009 | 51 (5.7%) | 43 (4.8%) | 0.458 |
| Obesity | 2,523 (21.2%) | 174 (17.7%) | 0.011 | 170 (19.2%) | 156 (17.6%) | 0.425 |
| Alcohol use | 32 (0.3%) | NA | 0.952 | NA | NA | 0.479 |
| Prior stroke | 714 (6.0%) | 122 (12.4%) | <0.001 | 98 (11.0%) | 101 (11.4%) | 0.880 |
| Myocardial infarction | 775 (6.5%) | 145 (14.7%) | <0.001 | 137 (15.4%) | 121 (13.6%) | 0.312 |
| Congestive heart failure | 4,762 (40.0%) | 743 (75.5%) | <0.001 | 653 (73.6%) | 657 (74.1%) | 0.871 |
| Peripheral vascular disease | 5,227 (43.9%) | 217 (22.1%) | <0.001 | 209 (23.6%) | 212 (23.9%) | 0.911 |
| Cerebrovascular disease | 775 (6.5%) | 95 (9.7%) | <0.001 | 89 (10.0%) | 86 (9.7%) | 0.873 |
| Dementia | 41 (0.3%) | 25 (2.5%) | <0.001 | 25 (2.8%) | 22 (2.5%) | 0.767 |
| Chronic pulmonary disease | 1,992 (16.7%) | 258 (26.2%) | <0.001 | 250 (28.2%) | 231 (26.0%) | 0.336 |
| Rheumatic disease | 50 (0.4%) | 8 (0.8%) | 0.127 | NA | NA | 1.000 |
| Peptic ulcer disease | 283 (2.4%) | 51 (5.2%) | <0.001 | 42 (4.7%) | 46 (5.2%) | 0.743 |
| Liver disease | 375 (3.1%) | 54 (5.5%) | <0.001 | 54 (6.1%) | 46 (5.2%) | 0.471 |
| Diabetes | 1,566 (13.1%) | 154 (15.7%) | 0.029 | 159 (17.9%) | 141 (15.9%) | 0.282 |
| Paraplegia | 152 (1.3%) | NA | 0.164 | NA | NA | 1.000 |
| Renal disease | 1,505 (12.6%) | 303 (30.8%) | <0.001 | 251 (28.3%) | 255 (28.7%) | 0.875 |
| Metastatic solid tumor | 31 (0.3%) | 11 (1.1%) | <0.001 | 11 (1.2%) | 11 (1.2%) | 1.000 |
| Charlson comorbidity index | 4.05 ±2.12 | 6.19 ±2.27 | <0.001 | 6.06 ±2.15 | 6.09 ±2.23 | 0.82 |

To express normally distributed continuous variables, we utilized the mean value ± standard deviation and conducted analysis via Student's t-test. Meanwhile, categorical variables were expressed numerically with percentages using either the X^2^ test or Fisher's exact test.

NA: mean <11 numbers are not reported according to the database suggestion.

Abbreviation: TAVR: transcatheter aortic valve replacement; SAVR: surgical aortic valve replacement; PCI: percutaneous coronary intervention; CABG: coronary artery bypass graft; PPM: permanent pacemaker implant; PVD: peripheral vascular disease.

* The median income in the ZIP code of each patient was stratified into quartile divisions annually, and thus categorized into groups representing low income (bottom 25%), medium income, high income, and the highest income (top 25%).

** The categorization of hospital sizes was predicated upon the total number of hospital beds, with designated cut-points being established for every combination of region and location. This was done in such a manner that roughly 1/3 of hospitals were classified within each size category.

Supplementary Table 3: The 30-day readmission reasons for pure AR patients undergoing the SAVR or TAVR in matching cohort.

| Reason | TAVR | SAVR | *P*-value |
| --- | --- | --- | --- |
| Arrhythmia | 30(14.8%) | 37(14.4%) | 0.36 |
| Heart failure | 38(18.7%) | 50(19.5%) | 0.17 |
| Permanent pacemaker implant | 32(15.8%) | NA | <0.01 |
| Intercranial hemorrhage | NA | NA | 0.66 |
| Myocardial infarction | NA | NA | 0.47 |
| Hypertension | NA | NA | 0.99 |
| Other cardiovascular | NA | 22(8.6%) | <0.01 |
| Valve disorder | NA | NA | 0.45 |
| Anemia | NA | NA | 0.78 |
| Cancer | NA | NA | 0.99 |
| Electrolyte abnormality | NA | NA | 0.12 |
| Infection | 13(6.4%) | 35(13.6%) | <0.01 |
| Gastrointestinal | 20(9.9%) | 20(7.8%) | 0.97 |
| Musculoskeletal | NA | NA | 0.98 |
| Respiratory | 10 (4.9%) | 28(10.9%) | <0.01 |
| Trauma | NA | NA | 0.49 |
| Diabetes | NA | NA | 0.67 |
| Neurological delirium seizure | NA | NA | 0.61 |
| Stroke | NA | NA | 0.98 |
| Surgical complication | NA | NA | 0.69 |
| Other non-cardiovascular | 12(5.9%) | 19(7.4%) | 0.2 |
| Peripheral artery disease | NA | NA | 0.04 |

Values are number with percentages using the Fisher’s exact test or the X^2^ test.

NA: mean <11 numbers are not reported according to the database suggestion.

Abbreviation: SAVR: surgical aortic valve replacement; AR: aortic regurgitation; TAVR: transcatheter aortic valve replacement.

Supplementary Table 4: The 6-month readmission reasons for pure AR patients undergoing the SAVR or TAVR in the second cohort after matching.

| Reason | TAVR | SAVR | *P*-value |
| --- | --- | --- | --- |
| Arrhythmia | 26(12.1%) | 29(11.3%) | 0.64 |
| heart failure | 40(18.6%) | 38(14.8%) | 0.96 |
| Permanent pacemaker implant | 25(11.6%) | NA | <0.01 |
| Intercranial hemorrhage | NA | NA | 0.36 |
| Myocardial infarction | NA | NA | 0.61 |
| Hypertension | NA | NA | 0.99 |
| Other cardiovascular | NA | 16(6.2) | 0.05 |
| Valve disorder | NA | NA | 0.57 |
| Anemia | NA | NA | 0.54 |
| Cancer | NA | NA | 0.69 |
| Electrolyte abnormality | NA | NA | 0.78 |
| Infection | 15(7.0%) | 24(9.3%) | 0.13 |
| Gastrointestinal | 26(12.1%) | 25(9.7%) | 0.92 |
| Musculoskeletal | 12(5.6%) | 15(5.8%) | 0.53 |
| Respiratory | NA | 27(10.5%) | <0.01 |
| Trauma | NA | NA | 0.49 |
| Diabetes | NA | NA | 0.43 |
| Neurological delirium seizure | NA | NA | 0.6 |
| Stroke | NA | NA | 0.98 |
| Surgical complication | NA | NA | 0.98 |
| Other non-cardiovascular | 13(6.0%) | 15(5.8%) | 0.65 |
| Peripheral artery disease | NA | NA | 0.33 |

Values are number with percentages using the Fisher’s exact test or the X^2^ test.

NA: mean <11 numbers are not reported according to the database suggestion.

Abbreviation: SAVR: surgical aortic valve replacement; AR: aortic regurgitation; TAVR: transcatheter aortic valve replacement.

Supplementary Figure 1: Data distribution before and after propensity matching for 30-day readmission


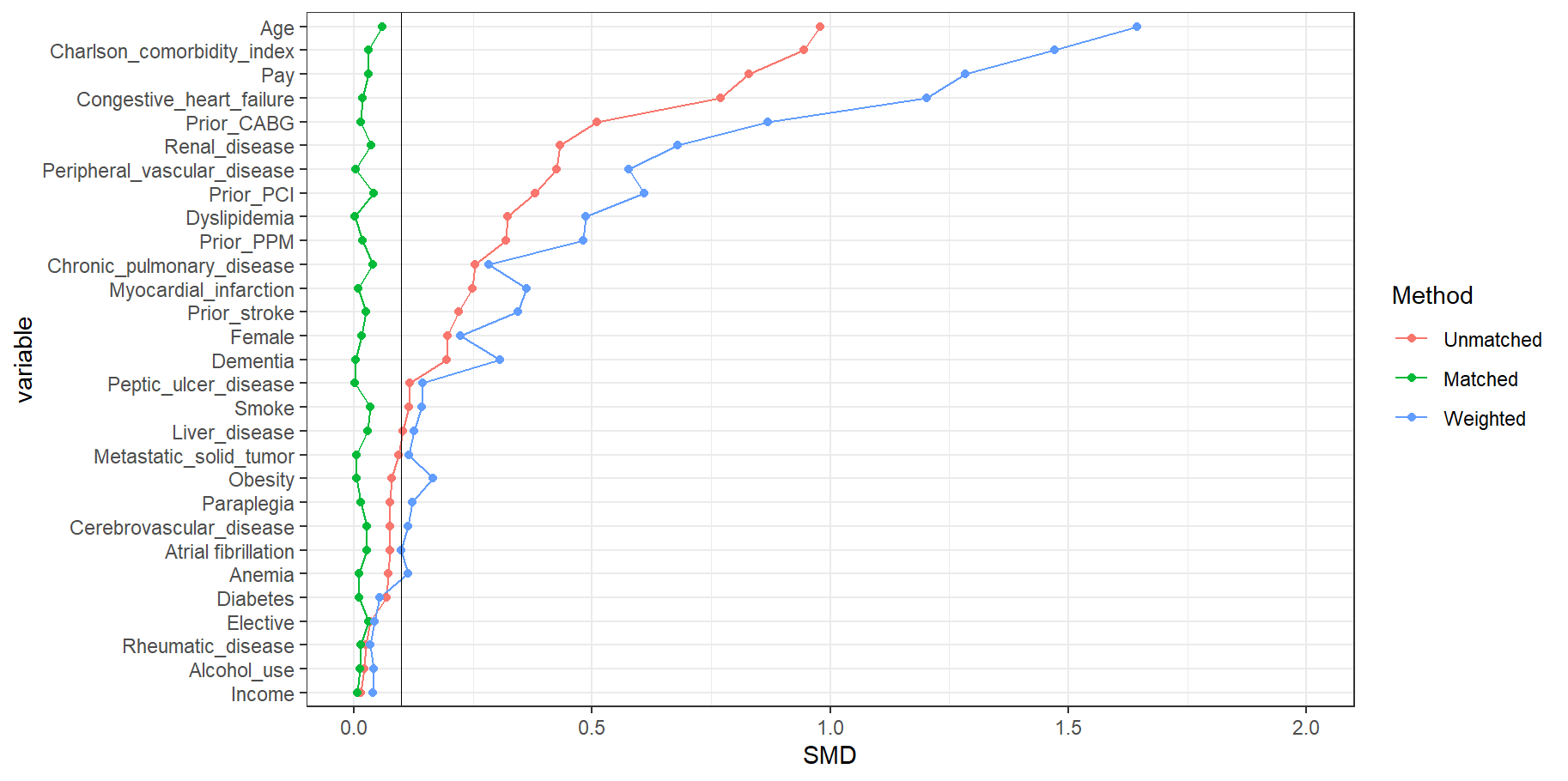


Abbreviation: SMD: standardized mean difference; PCI: percutaneous coronary intervention; PPM: permanent pacemaker implant; CABG: coronary artery bypass graft.

Supplementary Figure 2: Trends of AR patients receiving TAVR or SAVR between 2016 and 2019.

Abbreviation: SAVR: surgical aortic valve replacement; AR: aortic regurgitation; TAVR: transcatheter aortic valve replacement.

Supplementary Figure 3: Data distribution before and after propensity matching for second cohort.


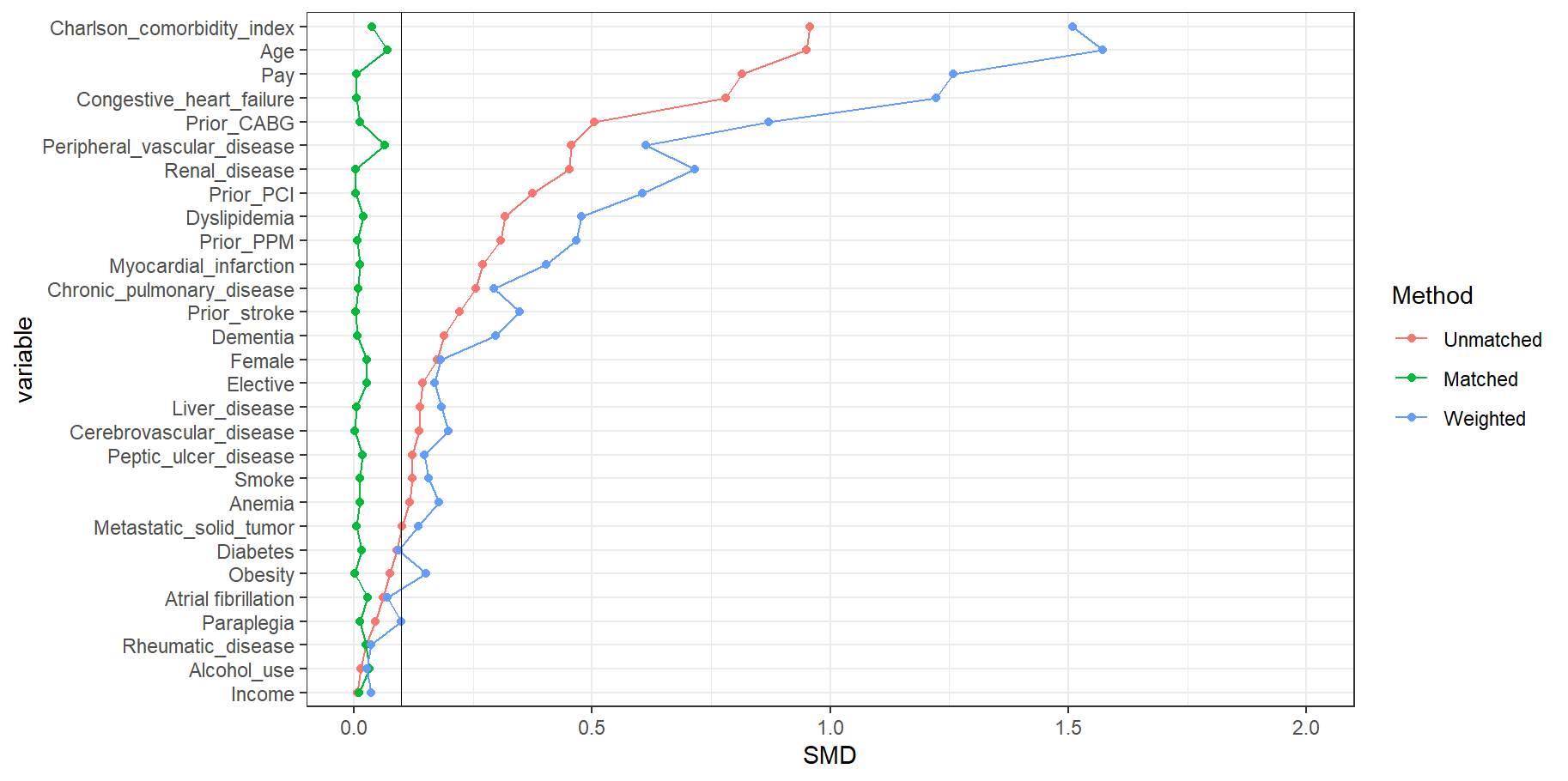


Abbreviation: SMD: standardized mean difference; PCI: percutaneous coronary intervention; PPM: permanent pacemaker implant; CABG: coronary artery bypass graft.

Supplementary Figure 4: Compared the surgical aortic valve replacement and transcatheter aortic valve replacement in the 30-day all-cause readmission, 6-month all-cause readmission incidence, 30-day permanent pacemaker implantation incidence and 6-month permanent pacemaker implantation incidence.


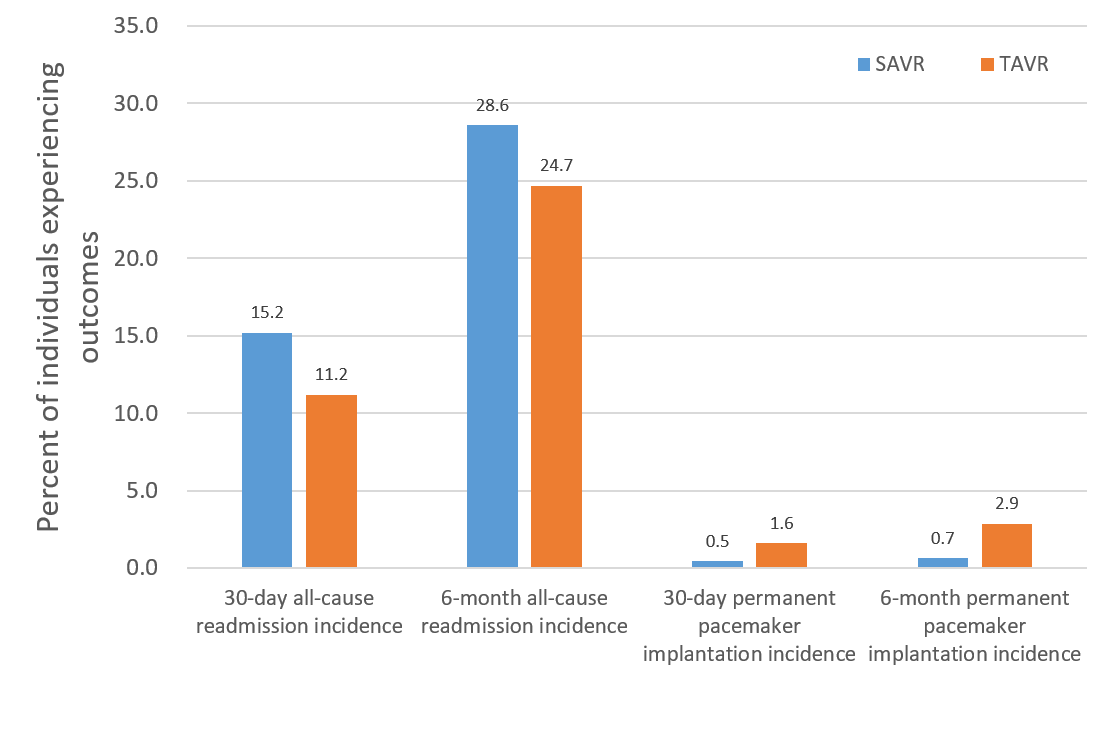

Supplement: Multimedia component 1 [file mmc1.docx]
